# Annual relative increased in inpatient mortality from antimicrobial resistant nosocomial infections in Thailand

**DOI:** 10.1017/S0950268818003436

**Published:** 2019-03-08

**Authors:** T. Phodha, A. Riewpaiboon, K. Malathum, P. C. Coyte

**Affiliations:** 1 Faculty of Pharmacy, Mahidol University, 447 Sri-Ayuthaya Road, Rajathevi, Bangkok 10400, Thailand; 2 Division of Infectious Disease, Department of Medicine, Faculty of Medicine Ramathibodi Hospital, Mahidol University, 270 Rama VI Road, Rajathevi, Bangkok 10400, Thailand; 3 Institute of Health Policy Management and Evaluation, School of Public Health, University of Toronto Ontario, Health Sciences Building 155 College Street, Suite 425 Toronto, ON, M5T 3M6, Canada

**Keywords:** Antibiotic resistance, health policy, hospital-acquired (nosocomial) infections, infectious disease epidemiology

## Abstract

Antimicrobial resistance is a major health threat worldwide as it brings about poorer treatment outcome and places economic burden to the society. This study aims to estimate the annual relative increased in inpatient mortality from antimicrobial resistant (AMR) nosocomial infections (NI) in Thailand. A retrospective cohort study was conducted at Ramathibodi Hospital, Bangkok, Thailand, over 2008–2012. Survival model was used to estimate the hazard ratio of mortality of AMR relative to those patients without resistance (non-AMR) after controlling for nine potential confounders. The majority of NI (73.80%) were caused by AMR bacteria over the study period. Patients in the AMR and non-AMR groups had similar baseline clinical characteristics. Relative to patients in the non-AMR group, the expected hazard ratios of mortality for patients in the AMR group with *Acinetobacter baumannii*, *Escherichia coli*, *Pseudomonas aeruginosa* and *Staphylococcus aureus* were 1.92 (95% CI 0.10–35.52), 1.25 (95% CI 0.08–20.29), 1.60 (95% CI 0.13–19.10) and 1.84 (95% CI 0.04–95.58), respectively. In the complete absence of AMR bacteria, this study estimated that annually, in Thailand, there would be 111 295 fewer AMR cases and 48 258 fewer deaths.

## Introduction

The discovery of antimicrobials was one of the most important medical advancements crucial to global health [1]. Antimicrobial resistance (AMR), partly related to the overuse of antimicrobial drugs, is now a common global health problem [2, 3]. Infections that were once easily cured with antimicrobial medications are now becoming more difficult, and in some cases impossible to treat effectively, resulting in premature mortality [4]. It was estimated that AMR could lead to 10 million deaths a year in 2050, globally [5]. Information regarding the relative increased in inpatient mortality due to AMR is useful to inform and motivate both change in clinical practice and policy decision making. Consequently, the purpose of this study was to estimate the relative increased in inpatient mortality from antimicrobial resistant nosocomial infections (AMR NI) in Thailand.

## Methods

A retrospective cohort study was conducted at Ramathibodi Hospital, Bangkok, a 1400-bed university hospital in Thailand, between 1 January 2008 and 31 December 2012. The population of this study were patients admitted to critical areas, namely, medical intensive care unit (MICU), intermediate medical care unit (IMCU), surgical intensive care unit (SICU), cardiovascular–thoracic surgical intensive care unit, and coronary care unit (CCU) who subsequently contracted NI in either critical areas or other wards. The patients in this cohort were identified by the infection control unit of Ramathibodi Hospital using the surveillance definitions of the United States Centers for Disease Control and Prevention (US-CDC) [6]. There was no randomisation process of sample selection. Patients were categorised based on the exposure of interest including AMR and non-AMR infections in order to estimate the relative increased in inpatient mortality of AMR to non-AMR [7, 8]. Patients who acquired infections caused by bacteria that were resistant to at least three classes of antimicrobial agents (multidrug-resistant, MDR) bacteria were allocated to the AMR group. Those who acquired sensitive bacteria were allocated to the non-AMR group. Patients with community-acquired infections and colonisation cases (as defined by infection prior to 2 days post-admission) were excluded. The five most common types of bacterial infections included in this study were *Acinetobacter baumannii*, *Escherichia coli*, *Klebsiella pneumoniae*, *Pseudomonas aeruginosa* and *Staphylococcus aureus* [9–11]. The three most common infection sites within the NI surveillance system were studied. These include pneumonia (PNI), urinary tract infection (UTI) and bloodstream infection (BSI) [9–11]. A Charlson co-morbidity index score (CCI score) was computed and assigned to each patient in order to adjust for his/her comorbidities and the severity of baseline diseases [12]. We censored the Cox model at hospital discharge. The survival time from date of infection until hospital discharge was captured by a time variable. The specimen collection date on which the symptoms were firstly noticed by the physician was chosen to represent the onset of NI [6]. The event of interest was inpatient mortality. A patient who died was treated as a failure case while a patient who was discharged alive was treated as a censored case. The hazard ratio of mortality was estimated using multivariate Cox proportional hazard model with STATA 15.0 controlling for a number of potential confounders. The certain covariates associated with inpatient mortality at *P* < 0.10 from the univariate analysis [13] were considered for backwards stepwise elimination method along with clinical considerations to construct the multivariate Cox proportional hazard model. The univariate analysis of survival function, the association among covariates, the nested model and the proportion hazard assumption (or Schoenfeld residuals) were tested [13]; see Supplementary Material for details (Supplementary Material is available on the Cambridge Core website).

The prevalence of AMR NI reported in published national surveillance [14] was used as a factor for estimating the number of AMR NI cases in a year. Then the expected annual number of deaths from AMR NI, overall and categorised by type of bacteria, was the product of the number of AMR NI cases in a year multiplied by the hazard ratios of mortality from the Cox model. The associated variance and the confidence bounds were computed to report the uncertainty of the extrapolated nationwide annual relative increased in inpatient mortality, see Supplementary Material for details (Supplementary Material is available on the Cambridge Core website).

This study protocol has been reviewed and approved by the Committee on Human Rights Related to Research Involving Human Subjects of Faculty of Medicine Ramathibodi Hospital, Mahidol University. The Institutional Review Board number for this study is ID 10-56-41. Data were anonymous, kept confidential and not linked to individuals.

### Hospital settings

At the time of study, the hospital had nine intensive care units covering medical, surgical and paediatric populations, as well as general inpatient care units. There were 10 infection control nurses and one physician running the infection prevention and control based on the US-CDC surveillance strategies. Infection prevention measures were mainly those recommended by the US-CDC, the Society for Healthcare Epidemiology of America and WHO. The infection prevention and control programme has been performing prospective NI surveillance focusing on ICU patients continuously for decades.

## Results

### Descriptive analysis

**Fig. 1.**
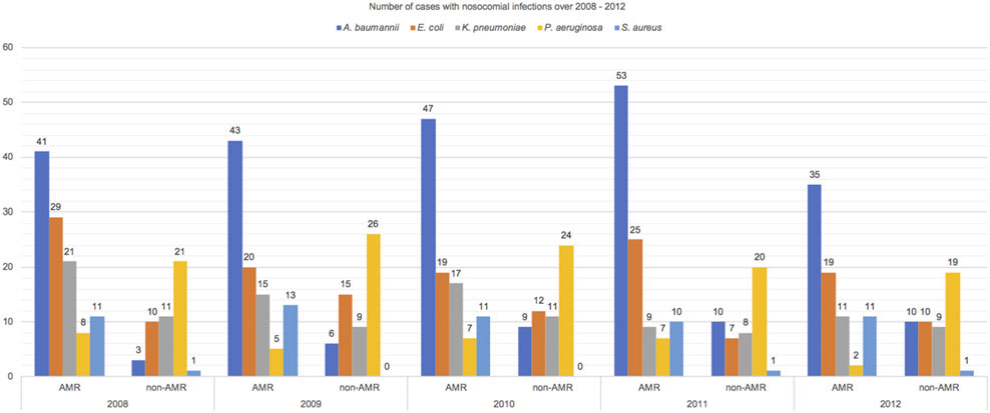
Number of cases with nosocomial infections over 2008–2012.

Study results were reported following STROBE guideline for an observational study [15]. There were 523 patients included in this cohort. Majority of NI during the study period (73.80%) were caused by the aforementioned five species of MDR organisms. Table 1 reports the distribution of patients’ characteristics. There was an almost equal mix of males and females and most of them were elderly. Higher proportion of patients with AMR infections than those with non-AMR infections (77% *vs.* 58%) in the MICU, IMCU and CCU were observed. The median length of hospital stay for patients in the AMR group was longer than those in the non-AMR group. The patients in both groups were different in the distribution of CCI score. Fifty per cent of patients acquired UTI, 30% acquired PNI, 15% acquired BSI, while the remaining patients acquired multiple site of infections. Among 523 critical care inpatients with NI, 201/386 (52%) patients with AMR and 53/137 (39%) with sensitive infections died. Figure 1 reports the number of cases with NI over 2008–2012. NI caused by *A*. *baumannii*, *E*. *coli*, *K*. *pneumoniae* and *S*. *aureus* had a greater proportion of AMR than non-AMR infections but *P*. *aeruginosa* had a smaller proportion of AMR than non-AMR infections.

**Table 1.** Descriptive analysis, *n* (%) or median (IQR)

Patient's characteristics	Susceptibility	
	AMR 386 (73.80)	Non-AMR 137 (26.20)
Gender		
Male sex	185 (47.93)	58 (42.34)
Age (years), median (IQR)	69 (56–81)	66 (48–79)
Admitted ward		
Surgical ICU	63 (16.32)	41 (29.93)
Cardiovascular–thoracic surgical ICU	26 (6.74)	17 (12.41)
Intermediate medical care unit	187 (48.45)	47 (34.31)
Coronary care unit	26 (6.74)	20 (14.60)
Medical ICU	84 (21.76)	12 (8.76)
LOS (days), median (IQR)	43 (27–67)	37 (24–65)
Mortality adjusted LOS (days), median (IQR)	51 (33–74)	39 (24–68)
Number of NI episode, mean ± s.d.	1.10 ± 0.31	1.01 ± 0.09
Charlson co-morbidity index score		
0	42 (10.88)	12 (8.76)
1	44 (11.40)	26 (18.98)
2	63 (16.32)	34 (24.82)
3	62 (16.06)	19 (13.87)
4	67 (17.36)	20 (14.60)
5	39 (10.10)	11 (8.03)
6	69 (17.88)	15 (10.95)
Site of infection		
UTI	174 (45.08)	71 (51.82)
Pneumonia	112 (29.02)	36 (26.28)
BSI	50 (12.95)	23 (16.79)
More than one site of infection	50 (12.95)	7 (5.11)
Discharge status		
Dead	201 (52.07)	53 (38.69)
Survive	185 (47.93)	84 (61.31)
Organisms^a^	489 (100)	254 (100)
* Acinetobacter baumannii*	219 (44.79)	38 (14.96)
* Escherichia coli*	112 (22.90)	55 (21.65)
* Klebsiella pneumoniae*	73 (14.93)	48 (18.90)
* Pseudomonas aeruginosa*	29 (5.93)	110 (43.31)
* Staphylococcus aureus*	56 (11.45)	3 (1.18)

a Multiple conditions.

### Survival analysis

Hazard ratios of inpatient mortality and 95% confidence bounds for covariates in the Cox proportional hazard regression model were reported in Table 2. Patients in the AMR group had a greater chance to die by 32% relative to those patients in the non-AMR group. There was no significant increase in the hazard of inpatient mortality related to AMR, for all tested bacteria. Hazard ratios were estimated to be 1.92 (95% CI 0.10–35.52), 1.25 (95% CI 0.08–20.29), 1.60 (95% CI 0.13–19.10) and 1.84 (95% CI 0.04–95.58) for *A. baumannii*, *E. coli*, *P. aeruginosa* and *S. aureus*, respectively. The other covariates adjusting for the Cox model comprised of sex, admitted ward, CCI score, site of infection, number of NI episode and the interaction effects between CCI score and site of infection.

**Table 2.** Multivariate cox proportional hazard regression model

Predictor variables	HR of in-hospital death	95% CI	*p*-value
Gender			
Female	1	1	
Male	1.23	0.95–1.59	0.129
Admitted ward			0.0001
Intermediate medical care unit	1	1	
SICU and cardiovascular–thoracic surgical ICU	0.65	0.47–0.91	0.012
Coronary care unit and medical ICU	1.42	1.06–1.92	0.020
CCI score			
0–2	1	1	
⩾3	1.42	0.87–2.32	0.000
Site of infection			0.1638
UTI	1	1	
Pneumonia	1.60	0.85–3.01	0.142
BSI	1.29	0.59–2.85	0.528
More than one site of infection	2.58	1.09–6.12	0.032
Number of NI episode	0.51	0.31–0.85	0.010
Susceptibility			
Non-AMR	1	1	
AMR	1.32	0.67–2.61	0.419
Organisms			0.4444
More than one organisms	1	1	
* A*. *baumannii*	0.69	0.23–2.07	0.509
* E*. *coli*	1.31	0.48–3.53	0.600
* K*. *pneumoniae*	1.35	0.52–3.47	0.536
* P*. *aeruginosa*	1.80	0.84–3.90	0.134
* S*. *aureus*	1.64	0.34–7.92	0.538
Susceptibility – organism			0.3190
Non-AMR	1	1	
AMR – *A*. *baumannii*	1.92	0.10–35.52	0.208
AMR – *E*. *coli*	1.25	0.08–20.29	0.568
AMR – *K*. *pneumoniae*	0.84	0.05–13.74	0.205
AMR – *P*. *aeruginosa*	1.60	0.13–19.10	0.442
AMR – *S*. *aureus*	1.84	0.04–95.58	0.845
Charlson co-morbidity score – site of infection			0.2971
CCI score = 0–2	1	1	
CCI score ⩾3 with pneumonia	1.55	0.57–21.76	0.217
CCI score ⩾3 with BSI	1.80	0.37–29.08	0.201
CCI score ⩾3 with more than 1 site of infection	0.80	0.30–28.22	0.635

### Nationwide annual relative increased in-hospital mortality from AMR NI in Thailand

The estimated nationwide annual relative increased in inpatient mortality from AMR NI in Thailand was reported in Table 3. This study estimated that around 111 295 AMR NI cases occurred in several types of hospitals in Thailand and 48 258 deaths in 2012. Resistant *A. baumannii* was the most common cause of death. About 60 000 cases with resistant *A. baumannii* infections accounted for almost 40 000 deaths in that year.

**Table 3.** Annual relative increased in-hospital mortality from AMR NI

(A) Annual number of AMR NI					
Bacteria	Proportion of NI	NI cases	Proportion of AMR NI	Variance of proportion of AMR NI	AMR NI cases
*Acinetobacter baumannii*	0.47	70 065	0.88	0.11	61 412
*Escherichia coli*	0.31	46 720	0.72	0.20	33 742
*Klebsiella pneumoniae*	0.23	34 022	0.73	0.20	24 796
*Pseudomonas aeruginosa*	0.25	37 777	0.57	0.24	21 628
*Staphylococcus aureus*	0.11	16 152	0.96	0.04	15 576
All AMR	1	150 806	0.74	0.19	111 295
(B) Annual number of deaths attributable to AMR NI					
Bacteria	Conditional mortality rate on having AMR	Variance of conditional mortality rate on having AMR	Overall AMR mortality	Variance of annual number of AMR mortality	95% CI of variance of annual number of AMR mortality
*Acinetobacter baumannii*	0.61	0.90	37 462	15 253	37 461–37 463
*Escherichia coli*	0.56	0.78	18 895	1153	18 895–18 896
*Klebsiella pneumoniae*	0.19	0.72	4712	773	4711–4713
*Pseudomonas aeruginosa*	1.40	0.61	30 279	1116	30 279–30 280
*Staphylococcus aureus*	1.49	1.62	23 208	1794	23 208–23 209
All AMR	0.32	0.13	48 258	4661	48 257–48 259

## Discussions

We found that almost three-quarters of all patients with NI acquired resistant bacterial infections over the study period. The number of cases with AMR NI in our study was consistent with findings in a recent study in Thailand [16]. The distribution of the type of bacterial infections was also in accordance with the national antibiogram data provided by the National Antimicrobial Resistance Surveillance Center Thailand (NARST) [17]. The relative increased in inpatient mortality from AMR NI was estimated using a survival analysis controlling for confounding factors [18–21], including CCI score in order to adjust patients’ clinical baseline as other recent studies had done [22–24]. Compared with patients in the non-AMR group, the mortality chances for patients in the AMR group with *A*. *baumannii*, *E*. *coli*, *P*. *aeruginosa* and *S*. *aureus* were 92%, 25%, 60% and 84% higher, respectively. Because resistant *E*. *coli* in our study was ESBL-producing organisms which can be treated by widely available carbapenem antimicrobials, mortality of infection caused by this bacteria remained low. Another explanation is that the most common site of *E. coli* infection was UTI, which generally is not significantly associated with fatal outcome. On the other hand, *A. baumannii* mostly causes infection of the respiratory system which tends to carry higher mortality background. Only a few antimicrobial agents such as colistin, a nephrotoxic agent and tigecycline, a bacteriostatic agent with unpromising efficacy in the literature, are available for treatment. As a result, mortality associated with *A. baumannii* infection in our study was high. There were several studies that reported the mortality chances of these bacterial infections in Thailand and worldwide. Lim *et al*. published in 2016 [16] estimated the mortality attributable to multidrug resistance in hospital-acquired infection in Thailand for *A*. *baumannii*, *E*. *coli*, *P*. *aeruginosa* and *S*. *aureus* as 70%, 19%, 65% and 44%, respectively. Yang *et al*. published in 2013 [23], a retrospective 10-year study was conducted at a 2900-bed teaching hospital located in Northern Taiwan, reported crude 14-day mortality rates of *A*. *baumannii* in hospital-acquired PNI and ventilator-associated PNI were 43.8% and 31.3%, respectively. Huang *et al*. published in 2012 [21], a retrospective cohort study was conducted in Taipei Veterans General Hospital, Taiwan, estimated odd ratio of mortality caused by carbapenem-resistant *A*. *baumannii* bacteraemia was 1.03 (95% CI 0.48–2.20) relative to carbapenem-susceptible *A*. *baumannii* bacteraemia. Abernethy *et al*. published in 2015 [25], a large national study in England, reported a 30-day all-cause mortality rate of *E*. *coli* was 18.2% (95% CI 17.8–18.7%). Schreiber *et al*. published in 2011 [19] reported the mortality rate in patients with bacteraemia complicated *S*. *aureus* PNI was 39% compared with non-bacteria. The meta-analysis of Cosgrove *et al*. published in 2003 [26], enrolled studies between January 1980 and December 2000 reported a significant increase in odd ratio (1.93; 95% CI 1.54–2.42; *P* < 0.001) of mortality associated with methicillin-resistant *S*. *aureus* bacteraemia compared with methicillin-susceptible *S*. *aureus* bacteraemia. Our study estimated higher expected hazard ratios of mortality for AMR NI relative to non-AMR NI than other studies. Overall, mortality of AMR NI in our study was not statistically significant different from those of non-AMR. It might be due to the fact that these patients were all critically ill which posed them at risk of dying. Furthermore, with relatively more available resources for treatment as compared with other non-teaching hospitals, mortality of AMR NI might be lowered as shown by some studies that effective antibiotics played an important role in improving outcomes of these infections [27, 28].

This study estimated that the annual relative increased in inpatient mortality in Thailand would be 48 258 cases out of 111 295 (over 50%) cases with AMR NI. The study of Lim *et al*. published in 2016 [16] estimated the burden of MDR bacteria in Thailand to be 19 122 of 45 209 (43%) deaths in 2010. The preliminary study of health and economic impact of AMR in Thailand published in 2012 [14] found 38 481 additional deaths of 87 481 (43%) AMR cases in 2010. However, our annual number of deaths is higher than those reported by others. We identified the annual number of deaths as the relative difference hazard ratio of mortality for the AMR and non-AMR groups based on the Cox model fitted with individual-level data of our study samples. To the best of our knowledge, this is the most updated data collected from patients in Thailand. The conditional mortality on having AMR was derived using the hazard ratio of mortality provided by the Cox model. Kritsotakis *et al*. in 2017 [11] also used the multivariate Cox proportional hazard model to estimate hazard of death between patients with hospital-acquired infections and uninfected patients in terms of adjusted hazard ratios and associated 95% confidence bounds. Even though, Lim *et al*. in 2016 [16] used a 30-day all-cause mortality rate while the preliminary study in 2012 [14] used a decision tree model. Moreover, our study took place in the critical care units of a large tertiary hospital. Most of the patients in our study had more severe illnesses than other hospital types. Consequently, the samples used may not necessarily be representative of the whole NI population in Thailand. This may result in an overestimate of AMR burden estimation for all hospitalised patients in the country. However, to the best of our knowledge, hospital-acquired infection surveillance in most hospitals in Thailand now focus on the ICU population according to targeted surveillance strategies.

## Limitations

There are four main study limitations comprising the selection of an appropriate comparator, the lack of adjustment for acute severity of illness parameters, the potential statistical challenges to the estimation and the lack of accounting for the competing risk of being discharged in the multivariate Cox proportional hazard regression model. First, we have taken the more conservative approach of assuming that resistant infections are replacing susceptible ones so that we used the susceptible ones as comparators. The majority of studies to date have compared outcomes in patients infected with the resistant strain of an organism with patients infected with the susceptible strain of the same organism [7]. We did not have patients with no infection as comparator to deal with the competing risk of infection (susceptible and resistant, which might differ) and death. Engemann *et al*. in 2003 [29] compared outcomes among patients with resistant infections to uninfected control subjects selected on the basis of specific criteria which assesses the burden of having a resistant infection rather than no infection. The latter type of comparison results in a much higher estimate of adverse events attributable to resistance [7]. Second, acute severity of illness scores and their use in predicting outcomes have gained considerable interest for predicting ICU and in-hospital mortality [30]. The data on the physiologic measurements for severity of illness score calculation were incomplete. The results of this study might overestimate the impact of AMR infection due to the lack of adjustment for acute severity of illness parameters. Third, the AMR status was treated as an independent variable in the multivariate Cox proportional hazard regression model. We did not address the endogeneity of AMR status. It is possible that variables correlated with AMR status may also be related to death. In the absence of controls for potential endogeneity bias, the estimated impact of AMR status on relative increased in inpatient mortality may be overestimated. Additionally, statistical power may not have been reached due to limited sample size for certain susceptibility group. Finally, in-hospital mortality was used, and therefore using Cox proportional hazards methods on such an outcome may have issues – such as not accounting for the competing risk of being discharged and overall mortality wherein mortality after discharge is included.

## Summary

AMR has become a major public health threat and is spreading worldwide. Infectious diseases are the second main cause of death in low- to middle-income countries. This study sought to estimate the relative increased in inpatient mortality from AMR NI in Thailand to non-AMR NI using a multivariate Cox proportional hazard regression model. The Cox modelling was controlled for nine potential confounders while the AMR group has an elevated risk of inpatient mortality by 32%, it is not significant. In the absence of AMR bacteria, this study estimated that annually there would be 111 295 fewer AMR cases and 48 258 fewer deaths. The findings will assist in informing policy decision makers in their enactment of Thailand's National Strategic Plan on Antimicrobial Resistance for 2017–2021.

## Supplementary material

For supplementary material accompanying this paper visit https://doi.org/10.1017/S0950268818003436. click here to view supplementary material
