# Supplementary material for: Annual relative increased in inpatient mortality from antimicrobial resistant nosocomial infections in Thailand
Source: Epidemiol Infect. 2019 Mar 8;147:e133. doi: 10.1017/S0950268818003436 (PMC6518492; doi:10.1017/S0950268818003436)
Supplement: Supplementary file 1 [file S0950268818003436sup001.doc]

*Epidemiology and Infection*

**Annual relative increased in-hospital mortality from antimicrobial resistant nosocomial infections in Thailand**

T. PHODHA, A. RIEWPAIBOON, K. MALATHUM, P.C. COYTE

**Data collection frame**


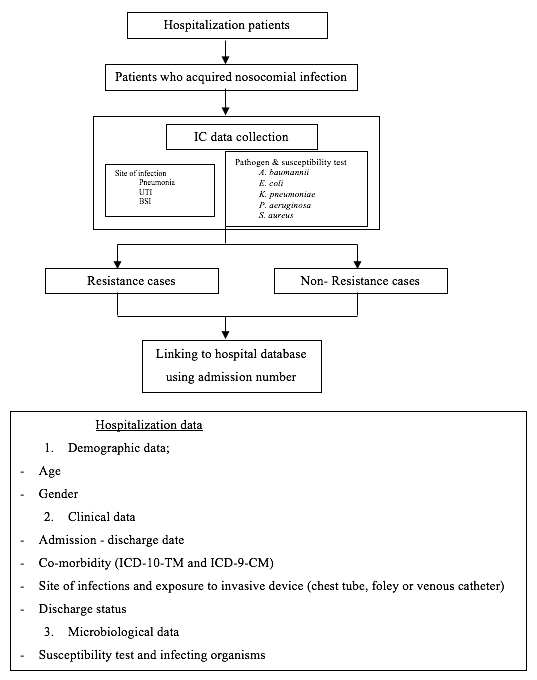


**Univariate analysis of survival function**

| **Variable** | | | **Death**  **(n)** | **Median survival time**  **(days)** | **Time at risk**  **(person-days)** | **Incidence rate**  **(100 person-days)** | **Hazard ratio**  **(95% CI)** | ***p-value*** |
| --- | --- | --- | --- | --- | --- | --- | --- | --- |
| Age (Years) | | |  |  |  |  | 1.00 (0.99, 1.00) | 0.7021 |
| Sex | | |  |  |  |  |  |  |
|  | | Female | 119 | 58 | 11,486 | 1.04 | 1 |  |
|  | | Male | 135 | 36 | 8,353 | 1.62 | 1.48 (1.16, 1.89) | 0.002 |
| Admitted ward | | |  |  |  |  |  | 0.5503 |
|  | IMCU | | 108 | 50 | 8,736 | 1.24 | 1 |  |
|  | SICU & CVTICU | | 62 | 66 | 6,772 | 0.92 | 0.77 (0.56, 1.05) | 0.096 |
|  | CCU & MICU | | 84 | 34 | 4,331 | 1.94 | 1.52 (1.14, 2.03) | 0.004 |
| CCI score | | |  |  |  |  |  |  |
|  | 0 - 2 | | 64 | 75 | 7,943 | 0.81 | 1 |  |
|  | ≥ 3 | | 190 | 38 | 11,896 | 1.60 | 1.90 (1.43, 2.53) | 0.000 |
| MDAI | | |  |  |  |  |  |  |
|  | Yes | | 45 | 43 | 3,350 | 1.34 | 1 |  |
|  | No | | 201 | 51 | 16,489 | 1.27 | 0.94 (0.68, 1.29) | 0.686 |
| Site of infection (SOI) | | |  |  |  |  |  |  |
|  | UTI | | 87 | 87 | 10,167 | 0.86 | 1 |  |
|  | Pneumonia | | 92 | 32 | 4,867 | 1.89 | 2.08 (1.55, 2.79) | 0.000 |
|  | BSI | | 42 | 38 | 2,168 | 1.94 | 2.10 (1.45, 3.04) | 0.000 |
|  | More than 1 SOI | | 33 | 53 | 2,637 | 1.25 | 1.37 (0.92, 2.05) | 0.123 |
| No. of NI episode | | |  |  |  |  | 0.62 (0.42, 0.92) | 0.019 |
| Susceptibility group | | |  |  |  |  |  |  |
|  | AMR | | 53 | 61 | 5,079 | 1.04 | 1 |  |
|  | Non-AMR | | 201 | 45 | 14,760 | 1.36 | 1.33 (0.98, 1.80) | 0.068 |
| Bacteria | | |  |  |  |  |  |  |
|  | More than 1 bacteria | | 75 | 53 | 7,569 | 0.99 | 1 |  |
|  | *A. baumannii* | | 96 | 31 | 5,155 | 1.86 | 1.74 (1.28, 2.36) | 0.000 |
|  | *E. coli* | | 23 | 107 | 2,773 | 0.83 | 0.72 (0.45, 1.16) | 0.175 |
|  | *K. pneumoniae* | | 18 | 65 | 1,921 | 0.94 | 0.92 (0.55, 1.54) | 0.743 |
|  | *P. aeruginosa* | | 30 | 42 | 1,915 | 1.57 | 1.40 (0.91, 2.14) | 0.123 |
|  | *S. aureus* | | 12 | 34 | 506 | 2.37 | 1.96 (1.06, 3.62) | 0.032 |

**Test for association among covariates**

|  | Sex | Susceptibility | No. of NI episode | CCI score | *A. baumannii* | *E. coli* | *K. pneumoniae* | *P. aeruginosa* | *S. aureus* | IMCU | SICU&CVTICU | CCU&MICU | UTI | PNI | BSI |
| --- | --- | --- | --- | --- | --- | --- | --- | --- | --- | --- | --- | --- | --- | --- | --- |
| Sex | 1.0000 |  |  |  |  |  |  |  |  |  |  |  |  |  |  |
| Susceptibility | 0.0493 | 1.0000 |  |  |  |  |  |  |  |  |  |  |  |  |  |
| No. of NI episodes | -0.0799 | 0.1334 | 1.0000 |  |  |  |  |  |  |  |  |  |  |  |  |
| CCI score | 0.0378 | 0.1171 | 0.0166 | 1.0000 |  |  |  |  |  |  |  |  |  |  |  |
| *A. baumannii* | 0.1298 | 0.2185 | -0.0990 | 0.0569 | 1.0000 |  |  |  |  |  |  |  |  |  |  |
| *E. coli* | -0.2190 | -0.0560 | -0.094 | -0.0668 | -0.2957 | 1.0000 |  |  |  |  |  |  |  |  |  |
| *K. pneumoniae* | -0.0173 | -0.0968 | -0.0796 | -0.0340 | -0.2035 | -0.1469 | 1.0000 |  |  |  |  |  |  |  |  |
| *P. aeruginosa* | 0.0313 | -0.3786 | -0.0893 | -0.0308 | -0.2282 | -0.1647 | -0.1134 | 1.0000 |  |  |  |  |  |  |  |
| *S. aureus* | 0.0531 | 0.0815 | -0.0525 | 0.0007 | -0.1341 | -0.0968 | -0.0666 | -0.0747 | 1.0000 |  |  |  |  |  |  |
| IMCU | -0.0518 | 0.1250 | 0.0125 | 0.0436 | -0.0763 | 0.0994 | 0.0203 | -0.0170 | -0.0353 | 1.0000 |  |  |  |  |  |
| SICU & CVTICU | 0.0316 | -0.1886 | 0.0136 | -0.1265 | -0.0723 | -0.1101 | -0.0514 | 0.0594 | -0.0251 | -0.5626 | 1.0000 |  |  |  |  |
| CCU & MICU | 0.0261 | 0.0508 | -0.0278 | 0.0791 | 0.1584 | 0.0002 | 0.0293 | -0.0410 | 0.0648 | -0.5493 | -0.3817 | 1.0000 |  |  |  |
| UTI | -0.1908 | -0.0595 | -0.1236 | 0.0038 | -0.1705 | 0.3915 | 0.1661 | 0.0286 | -0.1395 | 0.1956 | 0.2119 | -0.0045 | 1.0000 |  |  |
| PNI | 0.1722 | 0.0267 | -0.1419 | -0.0084 | 0.1681 | -0.2791 | -0.1409 | 0.0712 | 0.0587 | -0.1043 | 0.1643 | -0.0495 | -0.5898 | 1.0000 |  |
| BSI | 0.0452 | -0.0487 | -0.1009 | -0.0383 | 0.1432 | -0.0702 | 0.0440 | -0.0390 | 0.1905 | -0.1072 | 0.0305 | 0.0891 | -0.3781 | -0.2530 | 1.0000 |

**Test for collinearity among covariates**

We checked for collinearity among covariates. The VIF was equal to 1.41 means there was no collinearity problem among covariates in Cox model.

**Test for nested model between full model versus reduce model**

Covariates in full model comprise of sex, susceptibility group, number of NI episode, CCI score, type of bacteria, site of infection, admitted ward, interaction effect between susceptibility group – site of infection, susceptibility group – type of bacteria, cci score – site of infection, cci score – type of bacteria, and cci score – admitted ward

Covariates in reduced model based on stepwise backward elimination comprise of sex, susceptibility group, number of NI episode, CCI score, type of bacteria, site of infection, admitted ward, interaction effect between susceptibility group – type of bacteria and cci score – site of infection

Assumption reduced model based on the backwards stepwise elimination nested in full model

LR chi^2^ 7.37 df = 2

*p-value* = 0.6898

Interpretation: removing interaction effect between susceptibility group – site of infection, cci score – type of bacteria, and cci score – admitted ward together (not just individually) results in improvement in model fit.

**Test for proportional hazard assumption and Schoenfeld residuals**

There was no statistically significant (*p-value* = 0.6278) of the global test for proportional hazard assumption then we could not reject proportionality and consequently assumed that we did not have a violation of the proportionality assumption. We evaluated the goodness of fit of the Cox proportional hazard model by using the Cox-Snell residuals plot. See the Schoenfeld residuals plot for the global test of proportional hazard and the Cox-Snell residuals plot for overall goodness of fit test are shown in Figure A and B, respectively.

**Figure A** Schoenfeld residuals plot for the global test of proportional hazard

**
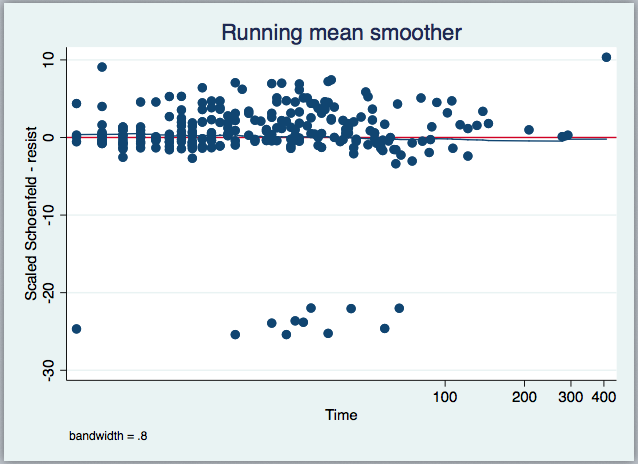
**

**Figure B** Cox-Snell residuals plot for overall goodness of fit test


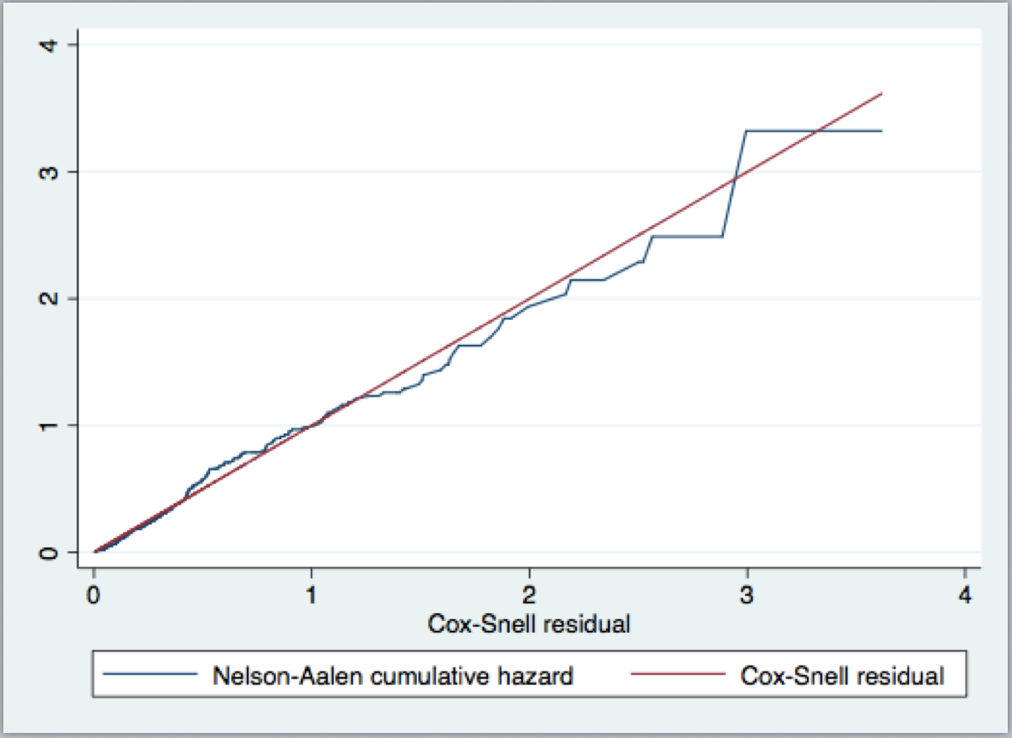


**Nationwide estimations for annual relative increased in-hospital mortality from AMR NI in Thailand**

This part reports the equations for computing the variance of overall AMR cases, the annual relative increased in-hospital mortality from AMR NI in Thailand including associated confidence intervals (CI).

1) Variance of AMR NI cases = se^2^ x n

2) Conditional mortality rate on having AMR = exp(β_bact)+exp(β_bact+β_resist+ β_bact_resist)

Note: Conditional mortality rate on having AMR means the relative risk or some hazard ratio of mortality for AMR vs susceptible NI.

3) Variance of conditional mortality rate on having AMR = exp(var_bact)+exp(var_bact +var_resist+2cov(bact,resist))

Note: The variance was from the covariance matrix of coefficiences of the Cox model.

4) AMR mortality = proportion of AMR x Conditional mortality rate on having AMR x Overall NI cases

5) Variance of annual number of AMR mortality = N^2^ x {(Incidence rate of AMR^2^ x Variance of conditional mortality rate on having AMR) + (Conditional mortality rate on having AMR^2^ x Variance of incidence rate of AMR) + (Variance of incidence rate of AMR x Variance of conditional mortality rate on having AMR)}

6) 95% CI of variance of annual number of AMR mortality = N ± 1.96 (sqrt(se))
